# Clinical characteristics and mortality risk factors in pediatric hypertrophic, restrictive, and rapidly progressive hypertrophic cardiomyopathy: a retrospective cohort study with follow-up

**DOI:** 10.3389/fcvm.2025.1541651

**Published:** 2025-03-31

**Authors:** Asad Nawaz, Zixian Sheng, Muhammad Junaid Akram, Jianjin Li, Lingjuan Liu, Yuxing Yuan, Jie Tian

**Affiliations:** Department of Cardiology, National Clinical Research Center for Child Health and Disorders, Ministry of Education Key Laboratory of Child Development and Disorders, China International Science and Technology Cooperation Base of Child Development and Critical Disorders, Chongqing Key Laboratory of Pediatric Metabolism and Inflammatory Diseases, Key Laboratory of Children’s Important Organ Development and Diseases of Chongqing Municipal Health Commission, National Clinical Key Cardiovascular Specialty, Children’s Hospital of Chongqing Medical University, Chongqing, China

**Keywords:** hypertrophic cardiomyopathy, restrictive cardiomyopathy, rapidly progressive hypertrophic cardiomyopathy, mortality risk factors, genetic testing

## Abstract

**Background:**

Pediatric cardiomyopathies are rare but life-threatening conditions with high mortality. Limited data exists on their clinical features and risk factors, especially in Asian populations, highlighting the need for further research in this area.

**Methods:**

This retrospective cohort study analyzed data from 212 pediatric patients diagnosed with hypertrophic cardiomyopathy (HCM), restrictive cardiomyopathy (RCM), or restrictive phenotype hypertrophic cardiomyopathy (RP-HCM) at a single center in China from October 2012 to October 2023, with follow-up until October 31, 2024. Demographic, clinical, and diagnostic data, as well as follow-up outcomes, were reviewed. Logistic and Cox regression models identified risk factors for in-hospital and long-term mortality.

**Results:**

Among the 212 patients, 79.72% (169/212) had HCM, 16.98% (36/212) had RCM, and 3.30% (7/212) had RP-HCM. Infection (75.47%, 160/212) and heart failure (51.42%, 109/212) were common comorbidities. In-hospital mortality was 5.19% (11/212), with follow-up mortality of 20.28% (43/212). The independent risk factors for mortality included left ventricular ejection fraction (LVEF), pulmonary hypertension, and low-density lipoprotein (LDL) levels (*P* < 0.05). Patients with RP-HCM showed the poorest outcomes, with a follow-up mortality rate of 42.86%. Only 10.4% (22/212) of patients underwent genetic testing, yet the positive detection rate was 63.7% (14/22).

**Conclusions:**

This study underscores the importance of early diagnosis, genetic testing, and integrated management in pediatric cardiomyopathies. LVEF, pulmonary hypertension, and LDL levels are critical prognostic factors, offering insights for risk assessment and management in affected children.

## 1 Introduction

Pediatric cardiomyopathy is a rare but life-threatening condition with an estimated prevalence of approximately 1 in 100,000 (1, 2). Despite its rarity, it poses significant risks to affected children, leading to long-term influences on cardiac and pulmonary function, growth, development, and overall quality of life. Among the various types, dilated cardiomyopathy (DCM) and hypertrophic cardiomyopathy (HCM) are more frequently observed and extensively studied. In contrast, restrictive cardiomyopathy (RCM), characterized by impaired ventricular filling and diastolic dysfunction, remains less understood due to its lower incidence (1–8). Recently, the emergence of a distinct subtype, hypertrophic cardiomyopathy with restrictive phenotype (RP-HCM), has garnered attention. RP-HCM presents with more severe clinical manifestations and a worse prognosis than typical HCM. However, due to its recent recognition and rarity, comprehensive data on RP-HCM remain limited, leaving significant gaps in our understanding of its clinical course and management strategies (9–13).

While recent studies have expanded the understanding of pediatric cardiomyopathy, there is still a lack of data specific to Asian populations, where genetic and environmental factors may influence disease characteristics and outcomes (1–15). To address this gap, we conducted a single-center retrospective cohort study in China to evaluate the clinical characteristics, outcomes, and mortality risk factors in children with HCM, RCM, and RP-HCM. By analyzing a multi-year dataset, this study aims to identify potential prognostic indicators to inform clinical decision-making and improve long-term outcomes for pediatric patients with these cardiomyopathies.

## 2 Methods

The observational study protocol was approved by the Ethical Committee of Chongqing Medical University (Date 2020/No 160-1). The parents of all participants provided informed consent. The data supporting this study's findings are available from the corresponding author upon reasonable request.

### 2.1 Patients

All hospitalized patients (aged <18 years) diagnosed with HCM, RCM, or RP-HCM from October 2012 to October 2023 were included in the study. Patients with comorbid malignant tumors or those with data missing for ≥20% were excluded. Follow-up records were established for each patient, and telephone follow-ups were conducted annually after discharge, with the follow-up period ending either upon the child's death or by October 31, 2024. Based on the follow-up results, the children were categorized as survival, death, or lost to follow-up.

### 2.2 Definition and diagnostic

The definition of CM in children is similar to that of adults. HCM refers to a thickening of the heart wall that cannot be explained by hemodynamic reasons and is not accompanied by ventricular dilation but physiological hypertrophy (such as that resulting from physical activity) and pathological hypertrophy (such as that resulting from hypertension, aortic valve stenosis, and other diseases) must be excluded. RCM is characterized by reduced ventricular compliance and is usually not associated with significant biventricular dilation, hypertrophy, or a decrease in systolic function. Their diagnostic criteria are based on pediatric guidelines (1, 2).

Research on RP-HCM in children is limited, and the diagnostic criteria refer to those for adults, including the following aspects: significant enlargement of both atria, mitral inflow E/A ratio of ≥2, and mitral deceleration time of ≤150 ms; absent or only mild left ventricular hypertrophy (LVH); ventricular cavity reduced or of normal size; left ventricular ejection fraction (LVEF) normal or mildly decreased (9–14).

### 2.3 Variables and outcomes

The primary outcome measure was all-cause mortality during the follow-up period, whereas the secondary outcome measure was in-hospital all-cause mortality. Data, including demographic characteristics, clinical features, auxiliary examinations, Genetic Report, diagnosis, and outcomes information, were collected for all patients based on their electronic medical records or clinical charts. All clinical characteristics were captured at the time of initial diagnosis/first evaluation at our center.

### 2.4 Statistical analyses

Data processing was performed using RStudio 4.4.1 software. Differences with *P* < 0.05 were considered statistically significant. Impute missing values using the random forest algorithm. The Kolmogorov–Smirnov test was applied to verify whether our data were normally distributed. Means and standard deviations were calculated to describe the variables for normally distributed data, while medians and ranges were calculated for non-normally distributed data. Frequencies and percentages were used for categorical data. Significant differences between the two groups were measured using *χ*^2^ or Fischer exact tests for categorical variables and the *t*-test or Wilcoxon rank-sum test for continuous variables.

Additionally, we analyzed the factors affecting in-hospital mortality risk using a logistic regression model and the factors affecting mortality risk during the follow-up period using a Cox regression model. Performed inter-group difference analysis and univariate regression analysis to screen influencing factors, and then explored independent risk factors through multivariate stepwise regression analysis.

## 3 Results

### 3.1 General characteristics

A total of 212 patients were included in the study, including 169 (79.72%) for HCM, 36 (16.98%) for RP-HCM, and 7 (3.30%) for RP-HCM. The median age at admission was 0.83 (0.33, 7.00) years, the male-to-female ratio was about 1.9:1, 52.36% of patients were admitted through outpatient clinics, and 70.75% were diagnosed with cardiomyopathy for the first time. Infection (75.47%) was the most common co-diagnosis, followed by heart failure (51.42%) and arrhythmia (20.28%). The average length of hospitalization was 12.09 ± 11.75 days, and 11 cases (5.19%) died of all causes in hospital. Two patients (1 with HCM and 1 with RP-HCM) underwent pacemaker implantation during hospitalization. The duration of follow-up was 4.24 (1.87, 7.61) years, and the mean age at the end of follow-up was 7.56 (3.74, 12.51) years. During follow-up, 43 (20.28%) cases died, and 11 (5.19%) cases were lost to follow-up (Table 1).

**Table 1:** Total sample characteristics and comparison of RCM, HCM, and RP-HCM.

Variables	Total (*n* = 212)	RCM (*n* = 36)	HCM (*n* = 169)	RP-HCM (*n* = 7)
Demographic characteristics				
Age, years	0.83 (0.33, 7.00)	4.00 (1.48,9.00)	0.67 (0.33,5.00)*	8.00 (5.00,11.50)
Sex				
Girl	74 (34.91%)	10 (27.78%)	61 (36.09%)	3 (42.86%)
Boy	138 (65.09%)	26 (72.22%)	108 (63.91%)	4 (57.14%)
Admission route				
Outpatient	111 (52.36%)	90 (53.25%)	16 (44.44%)	5 (71.43%)
Emergency	40 (18.87%)	34 (20.12%)	6 (16.67%)	NA
Transferred	61 (28.77%)	45 (26.63%)	14 (38.89%)	2 (28.57%)
Modified ROSS classification				
I–II	86 (40.57%)	13 (36.11%)	73 (43.20%)	NA
III–IV	126 (59.43%)	23 (63.89%)	96 (56.80%)	7 (100.00%)
Diagnostic-related				
Initial Diagnosis	150 (70.75%)	27 (75.00%)	121 (71.60%)	2 (28.57%)
Primary combined disease				
Infection	160 (75.47%)	24 (66.67%)	135 (79.88%)	1 (14.29%)
Heart Failure	109 (51.42%)	26 (72.22%)	76 (44.97%)*	7 (100.00%)
Arrhythmia	43 (20.28%)	7 (19.44%)	32 (18.93%)	4 (57.14%)
Auxiliary examination				
Electrocardiogram				
ST-T changes	83 (39.15%)	56 (33.14%)	23 (63.89%)*	4 (57.14%)
QT prolongation	48 (22.64%)	35 (20.71%)	10 (27.78%)	3 (42.86%)
High PR	18 (8.49%)	15 (8.88%)	2 (5.56%)	1 (14.29%)
Ventricular hypertrophy	66 (31.13%)	54 (31.95%)	10 (27.78%)	2 (28.57%)
Atrial Hypertrophy	42 (19.81%)	24 (14.20%)	15 (41.67%)*	3 (42.86%)
LVEF, %	62.34 ± 11.96	58.19 ± 10.26	63.50 ± 12.20*	55.71 ± 7.70
LVFS, %	33.90 ± 10.27	30.33 ± 6.49	34.88 ± 10.86*	28.57 ± 5.13
E/A ratio	1.37 ± 0.49	1.67 ± 0.54	1.31 ± 0.45*	1.30 ± 0.65
IVRT, s	69.42 ± 28.85	80.14 ± 25.72	66.25 ± 27.51*	90.71 ± 52.21
RVSP, mmHg	28.89 ± 14.16	36.78 ± 21.58	26.76 ± 10.56*	39.71 ± 23.89
Valvular regurgitation	123 (58.02%)	35 (97.22%)	81 (47.93%)*	7 (100.00%)
Pericardial effusion	39 (18.40%)	13 (36.11%)	25 (14.79%)*	1 (14.29%)
Pulmonary hypertension	42 (19.81%)	14 (38.89%)	24 (14.20%)*	4 (57.14%)
Outcomes				
Length of hospital stay, days	12.09 ± 11.75	14.53 ± 17.66	11.66 ± 10.30	10.00 ± 4.43
Hospitalization outcome				
Survival	201 (94.81%)	35 (97.22%)	160 (94.67%)	6 (85.71%)
Death	11 (5.19%)	1 (2.78%)	9 (5.33%)	1 (14.29%)
Duration of follow-up, years	4.24 (1.87, 7.61)	5.55 (1.94, 9.55)	4.27 (1.78, 6.76)*	2.35 (1.57, 2.89)
Age at the end of follow-up, years	7.56 (3.74, 12.51)	9.83 (7.36,13.95)	6.65 (3.40,11.95)*	8.80 (7.76,14.08)
Follow-up outcomes				
Death	43 (20.28%)	7 (19.44%)	33 (19.53%)	3 (42.86%)
Survival	158 (75.53%)	26 (72.22%)	129 (76.33%)	3 (42.86%)
Lost	11 (5.19%)	3 (8.33%)	7 (4.14%)	1 (14.29%)

HCM, hypertrophic cardiomyopathy; RCM, restrictive cardiomyopathy; RP-HCM, hypertrophic cardiomyopathy with restrictive phenotype; Transferred, transferred from other medical institutions group; LVEF, left ventricular ejection fraction; LVFS, left ventricular fractional shortening; IVRT, isovolumic relaxation time; RVSP, right ventricular systolic pressure.

* Comparison with RCM group, *P* < 0.05.

### 3.2 Types of cardiomyopathies

Because the sample size of PR-HCM was too small, we only compared the RCM group with the HCM group. The results showed that the median age of RCM children was significantly higher than that of the HCM group [4.00 (1.48, 9.00) years vs. 0.67 (0.33, 5.00) years], and there was no statistical difference in the sex ratio among the groups. More men than women. The proportion of children with heart failure in the RCM group was significantly higher than that in the HCM group (72.22% vs. 44.97%), and the proportion of children with co-infection in both groups was higher than 2/3 (RCM 66.67%; HCM 79.88%), about 1/5 of the patients with arrhythmia (RCM 19.44%; HCM 18.93%). The results of cardiac ultrasound showed that LVEF (58.19 ± 10.26% vs. 63.50 ± 12.20%) and LVFS (30.33 ± 6.49% vs. 34.88 ± 10.86%) in the RCM group were significantly lower than those in the HCM group. The E/A ratio of RCM group was (1.67 ± 0.54 vs. 1.31 ± 0.45), IVRT (80.14 ± 25.72 s vs. 66.25 ± 27.51 s) and RVSP (36.78 ± 21.58 mmHg vs. 26.76) ± 10.56 mmHg), pulmonary hypertension (97.22% vs. 47.93%), pericardial effusion (36.11% vs. 14.79%) and moderate to severe valve regurgitation (38.89% vs. 14.20%) were significantly higher than those in HCM group (Table 1). The results of electrocardiogram, cardiac biomarkers, complete blood count, and biochemical tests were different in only some indicators (Supplementary Table 1). All the above differences were statistically significant (*P* < 0.05).

There was no significant difference in outcome indicators between the HCM and RCM groups. The in-hospital all-cause mortality was 2.78% (1/36) in the RCM group and 19.44% (7/36) in the follow-up period. In-hospital all-cause mortality was 5.33% (9/169) in the HCM group and 19.53% (33/169) during follow-up (Table 1).

We also described the characteristics of the PR-HCM group, and the results showed that the median age of 7 children at admission was 8.00 (5.00, 11.50) years, all with heart failure, and the modified cardiac function grade was III to IV. Four patients (57.14%) had arrhythmia, but only one (14.29) had infection. LVEF (55.71 ± 7.70%) and LVFS (28.57 ± 5.13%) of these children were lower than the mean values of HCM and RCM groups, and the results of IVRT (90.71 ± 52.21) and RVSP (39.71 ± 23.89) were more similar to those of RCM group than HCM group. All seven patients had moderate to severe valve regurgitation, and four (57.14%) had pulmonary hypertension. Although only one (14.29%) person died during hospitalization in the PR-HCM group, all-cause mortality was 42.86% (3/7) during follow-up (Table 1).

### 3.3 Comparison of patients with different admission routes

111, 40 and 61 patients were transferred to outpatient, emergency and other medical institutions, respectively. There were no significant differences in age and sex ratio among the three groups. However, the modified ROSS classification of Transferred group and emergency group was III–IV (Transferred group 86.89%; 75% in the emergency group) and the first diagnosis of cardiomyopathy (85.25% in the Transferred group; The proportion of patients in the emergency group (77.50%) was significantly higher than that in the outpatient group (38.74% with modified ROSS grade III to IV, 60.36% with first test diagnosis). The proportion of patients with infection in the emergency department group was the highest (87.50%), followed by the Transferred group (80.33%) and the outpatient group (68.47%) (the above differences were statistically significant (*P* < 0.05, Table 2). No significant differences were observed in heart failure, arrhythmia, and laboratory test results among the three groups (Supplementary Material 3).

**Table 2:** Comparison of patients admitted through different modes of admission.

Variables	Outpatient (*n* = 111)	Emergency (*n* = 40)	Transferred (*n* = 61)
Demographic characteristics			
Age, years	1.33 (0.38,7.50)	0.46 (0.31,3.25)	0.83 (0.33,5.00)
Sex			
Girl	41 (36.94%)	13 (32.50%)	20 (32.79%)
Boy	70 (63.06%)	27 (67.50%)	41 (67.21%)
Modified ROSS classification			
I–II	68 (61.26%)	10 (25.00%)*	8 (13.11%)*
III–IV	43 (38.74%)	30 (75.00%)*	53 (86.89%)*
Diagnostic-related			
Initial Diagnosis	67 (60.36%)	31 (77.50%)*	52 (85.25%)*
Type of CM			
HCM	90 (81.08%)	34 (85.00%)	45 (73.77%)
RCM	16 (14.41%)	6 (15.00%)	14 (22.95%)
RP-HCM	5 (4.50%)	0 (0.00%)	2 (3.28%)
Primary combined disease			
Infection	76 (68.47%)	35 (87.50%)*	49 (80.33%)*
Heart failure	53 (47.75%)	20 (50.00%)	36 (59.02%)
Arrhythmia	26 (23.42%)	4 (10.00%)	13 (21.31%)
Outcomes			
Length of hospital stay, days	12.74 ± 12.79	10.40 ± 12.44	12.02 ± 9.08
Hospitalization outcome			
Survival	105 (94.59%)	38 (95.00%)	58 (95.08%)
Death	6 (5.41%)	2 (5.00%)	3 (4.92%)
Duration of follow-up, years	5.00 (2.52,7.41)	3.39 (0.74,5.03)	4.05 (0.50,8.83)
Age at the end of follow-up, years	8.43 (5.67,12.33)	5.32 (2.66,9.70)	8.26 (2.52,13.70)
Follow-up outcomes			
Death	14 (12.61%)	11 (27.50%)*	18 (29.51%)*
Survival	92 (82.88)	29 (72.50)*	37 (60.66)*
Lost	5 (4.50%)	NA	6 (9.84%)*

Transferred, transferred from other medical institutions group; HCM, hypertrophic cardiomyopathy; RCM, restrictive cardiomyopathy; RP-HCM, hypertrophic cardiomyopathy with restrictive phenotype.

* Comparison with outpatient group, *P* < 0.05.

Regarding outcomes, although there was no significant difference in in-hospital all-cause mortality among the three groups, all-cause mortality during follow-up was significantly higher in both the Transferred (29.51%) and emergency (27.50%) groups than in the outpatient group (12.61%). All the above differences were statistically significant (*P* < 0.05, Table 2).

### 3.4 Risk factors for death from cardiomyopathy in children

**Figure 1:**
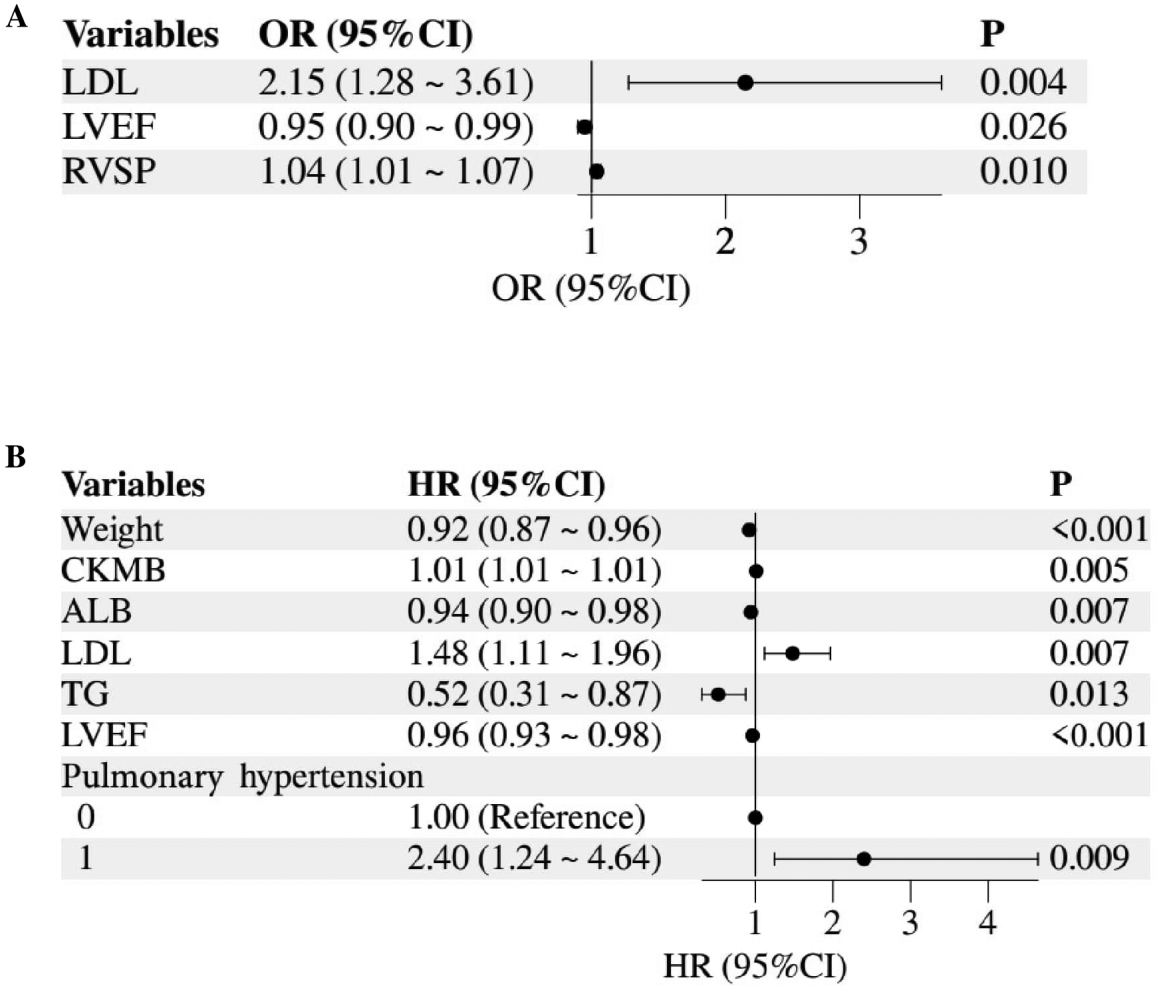
Forest plot of mortality risk factors in children with HCM or RCM. **(A)** Logistic regression for in-hospital mortality risk; **(B)** cox regression for mortality risk during follow-up. LDL, low-density lipoprotein; LVEF, left ventricular ejection fraction; RVSP, right ventricular systolic pressure; CKMB, creatine kinase MB; ALB, albumin; TG, triglycerides.

Logistic stepwise regression analysis showed that LDL (OR 2.15; 95% CI 1.28–3.61) and RVSP (OR 1.04; 95% CI 1.01–1.07) were positively correlated with the risk of hospital death in children with cardiomyopathy, LVEF (OR 0.95; 95% CI 0.90–0.99) was negatively associated with the risk of death in hospital (Figure 1A, Supplementary Material 5). COX stepped-regression analysis showed Pulmonary hypertension (OR 2.40; 95% CI 1.24–4.64), LDL (OR 1.48; 95% CI 1.11–1.96), CKMB (OR 1.01; 95% CI 1.01–1.01) was positively associated with the risk of death during follow-up in children with cardiomyopathy, while Weight (OR 0.92; 95% CI 0.88–0.96), ALB (OR 0.94; 95% CI 0.90–0.98), TG (OR 0.52; 95% CI 0.31–0.87), LVEF (OR 0.96; 95% CI 0.93–0.98) was negatively associated with the risk of death during follow-up (Figure 1B, Supplementary Material 6).

### 3.5 Genetic analysis

In our sample, only 10.4% (22/212) of the children were genetically tested. Of these 22, 68.2% (15/22) were tested after 5 years of age, and the positive detection rate was 63.7% (14/22). Among them, 40.1% (9/22) were pathogenic mutations, 27.3% (5/22) were likely pathogenic mutations. At the same time, 31.8% (7/22) had multiple gene mutations, and 40.1% (9/22) had mutations from their parents. The most frequently mutated gene was *MYH7* (5 HCM, 1 RCM and 3 RP-HCM), followed by *TNNI3* (1 HCM and 2 RCM) and *GAA* (2 HCM). Five children died during follow-up, two of whom had polygenic mutations, and the pathogenic or suspected mutations detected included *PTPN11* (1), *MYH7* (2), *TNNI3* (1), *KCNE3* (1), and *HCN4* (1) (Table 3).

**Table 3:** Genetic analysis.

NO.	Sex	Age at testing, years	Type of CM	Follow-up outcomes	Gene	Mutation	Variant type	Carrier
1	Boy	0.09	HCM	Survive	MYH7	Glu924Lys	LP	Mother
2	Girl	0.25	HCM	Survive	GAA	Pro424fs	P	Mother
					GAA	Glu888Ter	P	Father
3	Boy	0.33	HCM	Lost	DSP	GIn90Arg	VUS	Mother
					RBM20	Arg102Gln	VUS	Mother
					MYH6	Met1784Val	VUS	Mother
4	Girl	0.75	HCM	Survive	PKP2	Gly537Ala	VUS	Father
5	Boy	6.00	HCM	Death	PTPN11	Pro491Thr	P	None
6	Boy	7.00	HCM	Survive	MYH7	Leu863Pro	LP	None
7	Boy	8.00	HCM	Death	MYH7	Ala254Glu	VUS	None
8	Boy	12.00	HCM	Survive	LAMP2	Splicing	P	None
9	Girl	13.00	HCM	Survive	TNNI3	Arg186Gln	P	None
10	Boy	0.58	HCM	Survive	GAA	Ser601Leu	LP	Mother
					GAA	Trp481Arg	P	Father
11	Boy	13.00	HCM	Survive	MYH7	Arg783His	VUS	Father
12	Boy	6.00	HCM	Survive	MYBPC3	Glu258Lys	P	Mother
13	Boy	13.00	HCM	Survive	MYH7	Met822Va	P	None
14	Boy	0.08	RCM	death	Undetected	/	/	/
15	Boy	0.11	RCM	death	Undetected	/	/	/
16	Girl	5.00	RCM	Death	TNNI3	Arg192His	P	None
					PKP2	Ala749Asp	VUS	None
17	Boy	10.00	RCM	Survive	TNNI3	Arg192Cys	P	None
18	Boy	7.00	RCM	Death	MYH7	Leu863Pro	LP	None
19	Boy	5.00	RP-HCM	Survive	MYH7	Glu855del	LP	None
					BMPR2	Ala935Val)	VUS	None
					FHOD3	Ile1266Val	VUS	None
20	Girl	10.00	RCM	survive	Undetected	/	/	/
21	Girl	10.00	RP-HCM	Death	KCNE3	Thr4Ala	LP	Mother
					VCL	Asp735Asn	VUS	Father
					MYH7	Leu863Pro	LP	None
					TTN	Cys934Trp	VUS	Father
					HCN4	Ala195Val	VUS	Mother
22	Boy	13.00	RP-HCM	Lost	MYH7	Arg783His	VUS	Father
					TNNT2	Arg92Trp	VUS	Mother
					MYL3	Arg94His	VUS	Mother

HCM, hypertrophic cardiomyopathy; RCM, restrictive cardiomyopathy; RP-HCM, hypertrophic cardiomyopathy with restrictive phenotype. BMPR2, bone morphogenetic protein receptor type 2; DSP, desmoplakin; FHOD3, formin homology 2 domain containing 3; GAA, acid alpha-glucosidase; HCN4, hyperpolarization activated cyclic nucleotide gated potassium channel 4 Gene; KCNE3, potassium voltage-gated channel subfamily E regulatory subunit 3; LAMP2, lysosomal associated membrane protein 2; MYBPC3, cardiac myosin binding protein C; MYH6, alpha-myosin heavy chain; MYH7, beta-myosin heavy chain; MYL3, myosin light chain 3; PKP2, plakophilin; PTPN11, protein tyrosine phosphatase non-receptor type 11; RBM20, RNA binding motif protein 20; TNNI3, troponin I3, cardiac type; TNNT2, troponin T2, cardiac type; TTN, titin; VCL, vinculin. P, pathogenic; LP, likely pathogenic; VUS, variant uncertain significance.

## 4 Discussion

This study conducted long-term follow-up of hypertrophic cardiomyopathy (HCM), restricted cardiomyopathy (RP-HCM), and restricted phenotype hypertrophic cardiomyopathy (RP-HCM) in China, and comprehensively evaluated clinical features, outcomes, and mortality risk factors, emphasizing the importance of genetic testing, early diagnosis, and integrated management to improve prognosis. It enhances our current understanding of childhood cardiomyopathy.

Consistent with previous studies, far more children are diagnosed with HCM than RCM, and patients with HCM are also diagnosed at a younger age (3–8). Notably, the median age at HCM diagnosis in our cohort is younger than that reported in European/North American cohorts (7, 8), likely reflecting regional differences in early screening practices. Both cardiomyopathy phenotypes are more common in boys than in girls. Previous studies have suggested that RCM is rare in childhood, but the prognosis is poor (16). The results of our analysis showed that the heart function of children with RCM was significantly worse than that of HCM, specifically as follows: Children with RCM had lower left ventricular ejection fraction (LVEF) and left ventricular shortening fraction (LVFS) than children with HCM, and higher rates of early/atrial (E/A) ratio, isovolumic diastolic time (IVRT), right ventricular systolic blood pressure (RVSP), pulmonary hypertension, and moderate to severe valve regurgitation. Therefore, the incidence of heart failure on admission in the RCM group was significantly higher than that in the HCM group. Interestingly, despite these severe functional impairments, the cumulative mortality of RCM in our cohort (19.5%) was lower than historical Western data (5-year survival rate 51.1%–64.6%) (3, 17–19). This may be related to the fact that RCM children in our study were diagnosed at a younger age than previously reported, allowing timely intervention to delay disease progression. Additionally, the high infection comorbidity rate in our cohort [75.47% vs. <30% in Western studies (7)] underscores the need for region-specific strategies to address infectious triggers. In addition to the differences in the pathological mechanisms of the two phenotypes, this result may also be influenced by the generally late age of diagnosis of RCM. In addition, the progress of the medical level and the update of guidelines also provided great help to improve the clinical outcome of children with CM.

Of course, as we expected, children with this particular phenotype of RP-HCM had a very bad outcome. This is why the clinical characteristics and outcomes of children with RP-HCM are described separately (9–13). RP-HCM was first proposed by Kubo et al. (9), and restrictive filling pattern (10, 11) and restrictive physiology (12) were also named in other literature. Previous studies have mainly been carried out in adults. As one of the adverse remodeling types of HCM, its incidence in HCM ranges from 1.5% to 16% (9–11). Compared with typical HCM, RP-HCM has a more severe clinical phenotype, higher rates of atrial fibrillation, stroke, and heart failure, and a relatively poor clinical prognosis, with 22.6%–43.6% of patients dying or having a heart transplant during 5–10 years of follow-up (9–11). Of our patients, all seven had heart failure and moderate to severe valvular regurgitation, 57.14% had arrhythmias, and all of the cardiac ultrasound assessments were worse than those in the typical HCM group. Although only one person died during hospitalization, the all-cause mortality rate for RP-HCM was 42.86% during 2.35 (95% CI 1.57–2.89) years of follow-up. Although we only have seven samples, these results suggest that we need to pay attention to this particular phenotype and conduct higher-quality clinical and mechanistic studies in children to inform the development of treatment strategies in this population.

From the perspective of admission routes, nearly 50% of hospitalized children with cardiomyopathy are still from the emergency department or transferred hospitals (transferred from lower-level hospitals), and these children have worse heart function. The proportion of patients with first diagnosis of cardiomyopathy (77.5%–85.3%), co-infection (80.3%–87.5%), and mortality during follow-up (27.5%–29.5%) were higher than those admitted through outpatient clinics. Combined with the patient records of these children, we found that many children are still treated for heart failure caused by infection or infection, and then diagnosed with cardiomyopathy, but most of the children are at high risk of worsening heart function. This may be related to the lack of awareness of childhood cardiomyopathy and the imperfect management of early screening and diagnosis of childhood cardiomyopathy. Secondly, the high proportion of co-infection is also very worthy of attention, not only for cardiomyopathy. In all cases, infection is an important risk factor leading to the deterioration of children's cardiopulmonary function, so for children with cardiomyopathy, it is very important to do a good job of infection prevention and standardized treatment. Finally, for children with confirmed cardiomyopathy, regular outpatient follow-up and daily management are very important for delaying the course of the disease and improving the prognosis of the disease.

In addition, in our data, the median age of hospitalized children is less than 1 year old, and they can get reasonable diagnosis and treatment earlier, which reflects the progress of medical treatment level and is a very good phenomenon. However, the bad thing is that only 10.4% of the children had genetic testing, and among the children who had genetic testing, more than 2/3 of the sick children were older than 5 years old, which suggests that the coverage of genetic testing for children with cardiomyopathy may be insufficient, and the detection time is lagging. Whether in the study of adult or childhood cardiomyopathy, scholars have repeatedly emphasized the important value of genetic testing in cardiomyopathy (1–3, 20–23). Results of a multi-centre study conducted at 14 institutions in North America showed that 81 children tested for clinical cardiomyopathy gene combinations (21), 39 (48%) were positive and 19 (24%) were VUS of unknown significance, and the positive rate of testing was higher in children with HCM (68%) than in children with DCM. Therefore, genetic testing can help a large proportion of children with cardiomyopathy to make a definitive molecular genetic diagnosis. In addition to assisting in diagnosing and classifying cardiomyopathy, genetic testing is also an important basis for assessing prognosis and developing individualized treatment plans. For example, in HCM, patients with MYH7 gene mutation may have more obvious clinical symptoms, a younger age of onset, and a worse prognosis. Patients with MYBPC3 gene mutation showed more benign disease progression without serious clinical symptoms, and most of the first onset age was 40–50 years old (24). However, the clinical practice of genetic detection of childhood cardiomyopathy and the mechanism exploration of abnormal genes and clinical phenotypes still have many shortcomings that need improvement. Therefore, in clinical work for children with cardiomyopathy and children with first-degree family members with cardiomyopathy, we suggest actively improving genetic testing and genetic counselling (1, 2, 23), and we need to refer to the results of genetic testing to carry out more in-depth mechanism research to provide a reference for the precise treatment of cardiomyopathy.

Finally, we believe that LVEF, pulmonary hypertension, and LDL are all high-risk factors for death in children with HCM and RCM (25–30). LVEF is closely related to the heart's pumping function, especially in cardiomyopathy, and the decline of LVEF may mean the progressive deterioration of cardiac remodeling, accompanied by the progression of heart failure, resulting in adverse outcomes. RVSP is a measure of pulmonary hypertension, which may be caused by abnormalities in the structure and function of the heart and may aggravate the process of heart failure and ventricular remodeling; and is also considered to be an important factor indicating poor prognosis in children with cardiomyopathy (31). The effect of LDL on the prognosis of children with cardiomyopathy may be related to lipid metabolism, but its specific mechanism of action remains to be explored. We speculate that elevated LDL may reflect impaired myocardial energy metabolism or genetic defects in lipid regulation, as seen in other pediatric metabolic disorders. For example, a 2007 trial reported that omega-3 supplementation improved ventricular function in pediatric dilated cardiomyopathy, though LDL was not analyzed in that study (32). This suggests potential interactions between lipid homeostasis and myocardial repair. Previous scholars have suggested that cardiomyopathy and heart failure with ejection fraction retention can be regarded as metabolic diseases. Sodium-dependent glucose transporters 2 (SGLT-2) also improved cardiac function in these patients (33). On the other hand, recent studies in adults have found that either too low or too high LDL is strongly associated with an increased risk of death from cardiovascular disease. In our study, LVEF, pulmonary hypertension, and LDL are closely related to the risk of death in children with cardiomyopathy during hospitalization and post-discharge follow-up. These variables are easily accessible in clinical practice, and their combined use may enhance risk stratification. Future large-scale cohort studies are needed to validate their prognostic value and explore the underlying mechanisms through basic research.

The main limitation of this study was its retrospective, single-center design with a small sample size and limited genetic testing data, which may not have fully represented the characteristics of pediatric HCM and RCM. Despite this, long-term follow-up was conducted, and the clinical outcomes of RP-HCM were specifically described. Non-medical factors, including maternal history and education of guardians, showed no significant correlation with outcomes (SM1-4). Future multicenter studies with larger cohorts are warranted to advance the understanding of pediatric cardiomyopathy, particularly focusing on distinct subtypes such as HCM, RCM, and RP-HCM.

## 5 Conclusion

This study describes the clinical features, outcomes, and influencing factors of children with HCM, RCM, and RP-HCM in China. It emphasizes the importance of early diagnosis, genetic testing, and integrated management to improve prognosis. LVEF, pulmonary hypertension, and LDL levels are suggested as key risk factors for poor prognosis in children with cardiomyopathy, providing new insights for risk assessment and treatment management of pediatric cardiomyopathy.

## Data Availability

The original contributions presented in the study are included in the article/Supplementary Material, further inquiries can be directed to the corresponding authors.
