# Supplementary material for: Clinical characteristics and mortality risk factors in pediatric hypertrophic, restrictive, and rapidly progressive hypertrophic cardiomyopathy: a retrospective cohort study with follow-up
Source: Front Cardiovasc Med. 2025 Mar 31;12:1541651. doi: 10.3389/fcvm.2025.1541651 (PMC11994607; doi:10.3389/fcvm.2025.1541651)
Supplement: Supplementary file 1 [file Datasheet1.pdf]

Supplementary Table 1. Total Sample Characteristics and Comparison of RCM, HCM, and RP-HCM (Supplement)

Variables	Total (n = 212)	HCM (n = 169)	RCM (n = 36)	RP-HCM (n = 7)
Echocardiography				
LVEDD, mm	32.1 ± 7.3	33.1 ± 7.2	24.2 ± 7.4*	48.6 ± 9.4
RVEDD, mm	12.3 ± 3.5	12.6 ± 3.4	10.5 ± 3.7*	14.8 ± 4.4
LVPW, mm	9.1 ± 4.7	/	8.6 ± 4.3	11.6 ± 6.3
IVS, mm	11.6 ± 6.3	/	11.5 ± 6.4	12.3 ± 6.0
LA, mm	33.7 ± 6.9	33.7 ± 6.9	/	34.3 ± 7.9
RA, mm	33.7 ± 6.6	33.7 ± 6.5	/	34.3 ± 8.5
Outflow obstruction	24.10%	48 (28.4%)	/	3 (42.9%)
Cardiac Biomarkers				
BNP, pg/ml	1360.82 ± 3839.33	1358.11 ± 4243.28	1170.50 ± 1201.74	2405.01 ± 2075.06
CKMB, pg/ml	70.82 ± 435.64	52.03 ± 400.43	171.09 ± 601.35	8.72 ± 8.04
cTnI,ug/L,	1.69 ± 9.69	2.09 ± 10.82	0.11 ± 0.18	0.27 ± 0.40
Complete Blood Count				
RBC, 10^12/L	4.34 ± 0.68	4.32 ± 0.68	4.41 ± 0.65	4.65 ± 1.00
WBC, 10^9/L	10.67 ± 5.36	10.50 ± 5.20	11.58 ± 6.22	10.15 ± 4.76
Hb, g/L	115.39 ± 18.45	115.78 ± 18.48	112.19 ± 15.79	122.57 ± 28.81
PLT, 10^9/L	339.80 ± 153.92	356.15 ± 162.03	270.78 ± 96.88	299.86 ± 72.90
MCV, fl	82.88 ± 6.65	83.29 ± 6.77	80.57 ± 5.86	84.80 ± 5.30
MCH, pg	26.64 ± 2.52	26.94 ± 2.45	25.38 ± 2.51	26.07 ± 2.50
MCHC, g/L	322.37 ± 15.50	324.66 ± 15.15	313.53 ± 14.23	312.57 ± 10.92
Biochemical Tests				
ALT, U/L	90.15 ± 346.87	102.55 ± 387.24	41.09 ± 39.94	43.13 ± 41.49
AST, U/L	112.64 ± 217.58	124.93 ± 239.64	69.04 ± 77.18	40.16 ± 18.58
GTP, U/L	56.02 ± 72.64	47.84 ± 57.21	98.19 ± 117.20	36.63 ± 28.30
ALB, g/L	40.42 ± 6.58	40.98 ± 6.30	38.21 ± 7.48	38.50 ± 6.64
LDL,mmol/L	2.28 ± 1.00	2.27 ± 1.03	2.41 ± 0.91	1.82 ± 0.80
HDL, mmol/L	1.00 ± 0.39	1.01 ± 0.40	0.99 ± 0.36	0.86 ± 0.31
TG,mmol/L	1.36 ± 0.74	1.37 ± 0.73	1.38 ± 0.85	0.96 ± 0.28
ChE, U/L	6286.79 ± 1871.78	6299.52 ± 1853.08	6230.00 ± 2004.17	6271.57 ± 1898.74
Cr, umol/L	33.54 ± 14.98	32.83 ± 16.22	35.72 ± 8.35	39.47 ± 6.25
Sodium, mmol/L	138.86 ± 3.92	139.04 ± 3.94	138.02 ± 3.93	138.86 ± 3.26
Potassium, mmol/L	4.42 ± 0.69	4.46 ± 0.70	4.27 ± 0.60	4.23 ± 0.87
PT, s	18.88 ± 6.09	19.04 ± 6.67	18.32 ± 2.96	17.93 ± 1.88
APTT, s	16.57 ± 8.57	13.44 ± 5.31	28.23 ± 8.15	32.27 ± 5.41

SBP, Systolic Blood Pressure; DBP, Diastolic Blood Pressure; LVEDD left ventricle end diastolic
dimension; RVEDD right ventricle end diastolic dimension; LVPW left ventricle posterior wall; IVS intraventricular septum; LA left atria;RA right atria; BNP, B-type Natriuretic Peptide; CKMB, Creatine Kinase MB; cTnI, Cardiac Troponin I; RBC, Red Blood Cells; WBC, White Blood Cells; Hb, Hemoglobin; PLT, Platelets; MCV, Mean Corpuscular Volume; MCH, Mean Corpuscular Hemoglobin; MCHC, Mean Corpuscular Hemoglobin Concentration; ALT, Alanine Aminotransferase; AST, Aspartate Aminotransferase; GTP, Gamma-Glutamyl Transpeptidase; ALB, Albumin; LDL, Low-Density Lipoprotein; HDL, High-Density Lipoprotein; TG, Triglycerides; ChE, Cholinesterase; Cr, Creatinine; PT, Prothrombin Time; APTT, Activated Partial Thromboplastin Time.

Supplementary Table 2. Comparison of Patients Admitted Through Different Modes of Admission (Supplement)

Variables	Outpatient (n = 111)	Emergency (n = 40)	Transferred (n = 61)
**Demographic Characteristics**			
Mode of delivery			
Normal delivery	58 (52.25%)	13 (32.50%) *	38 (62.30%) #
Cesarean section	53 (47.75%)	27 (67.50%) *	23 (37.70%) #
Caregiver			
Others	8 (7.21%)	1 (2.50%)	5 (8.20%)
Parents	103 (92.79%)	39 (97.50%)	56 (91.80%)
Caregiver's education			
High school and below	86 (77.48%)	27 (67.50%) *	54 (88.52%) #
University and above	25 (22.52%)	13 (32.50%)	7 (11.48%)
**Clinical features**			
Weight, kg	15.78 ± 13.58	13.64 ± 14.98	14.44 ± 11.61
Height, cm	88.44 ± 33.91	78.36 ± 33.37	85.75 ± 33.81
Respiration, bpm	36.44 ± 14.14	45.15 ± 15.30*	39.38 ± 13.98
Heart rate, bpm	118.07 ± 26.95	131.97 ± 32.21*	124.97 ± 30.79
Blood pressure			
SBP, mmHg	98.70 ± 15.89	95.30 ± 14.29	100.90 ± 18.03
DBP, mmHg	58.98 ± 13.51	59.92 ± 10.00	60.23 ± 15.89
**Auxiliary examination**			
Electrocardiogram			
ST-T changes	37 (33.33%)	21 (52.50%)	25 (40.98%)
QT prolongation	26 (23.42%)	6 (15.00%)	16 (26.23%)
High PR	10 (9.01%)	4 (10.00%)	4 (6.56%)
Ventricular hypertrophy	32 (28.83%)	12 (30.00%)	22 (36.07%)
Atrial Hypertrophy	24 (21.62%)	6 (15.00%)	12 (19.67%)
Echocardiography			
LVEF, %	64.65 ± 11.51	58.10 ± 14.09*	60.92 ± 10.36#
LVFS, %	34.86 ± 8.26	30.52 ± 8.45	34.36 ± 13.79
E/A ratio	1.35 ± 0.49	1.43 ± 0.41	1.38 ± 0.53
IVRT, s	70.42 ± 28.66	68.78 ± 26.11	68.02 ± 31.21
RVSP, mmHg	28.01 ± 14.98	31.88 ± 15.62	28.52 ± 11.30
Valvular Regurgitation	64 (57.66%)	22 (55.00%)	37 (60.66%)
Pericardial effusion	14 (12.61%)	10 (25.00%)	15 (24.59%)
Pulmonary hypertension	18 (16.22%)	12 (30.00%)	12 (19.67%)
Cardiac Biomarkers			
BNP, pg/ml	1138.95 ± 3632.06	2428.30 ± 5977.72	1064.56 ± 1866.99
CKMB, pg/ml	37.63 ± 310.12	7.79 ± 6.63	172.54 ± 689.81
cTnI,ug/L,	0.99 ± 6.05	0.90 ± 2.49	3.50 ± 15.95
Complete Blood Count			
RBC, 10^12/L	4.36 ± 0.60	4.33 ± 0.77	4.33 ± 0.78

WBC, 10^9/L	10.88 ± 5.44	10.31 ± 5.51	10.53 ± 5.19
Hb, g/L	117.87 ± 16.48	115.10 ± 19.51	111.07 ± 20.52
PLT, 10^9/L	347.96 ± 160.88	323.95 ± 121.53	335.33 ± 161.08
MCV, fl	83.30 ± 6.62	83.69 ± 6.43	81.56 ± 6.75
MCH, pg	26.94 ± 2.39	26.68 ± 2.45	26.08 ± 2.73
MCHC, g/L	326.11 ± 14.51	318.32 ± 14.15*	318.21 ± 16.58*
Biochemical Tests			
ALT, U/L	60.31 ± 68.50	68.46 ± 55.58	158.69 ± 637.00
AST, U/L	81.45 ± 92.08	134.43 ± 168.29	155.09 ± 358.59
GTP, U/L	52.40 ± 77.51	49.91 ± 41.18	66.61 ± 79.23
ALB, g/L	41.75 ± 7.04	39.90 ± 5.30*	38.36 ± 5.95*
LDL, mmol/L	2.38 ± 1.08	1.96 ± 0.76	2.31 ± 0.97
HDL, mmol/L	1.01 ± 0.43	1.01 ± 0.35	0.98 ± 0.34
TG, mmol/L	1.36 ± 0.70	1.36 ± 0.82	1.34 ± 0.77
ChE, U/L	6612.77 ± 1975.97	5822.73 ± 1881.16*	5997.92 ± 1561.27*
Cr, umol/L	34.35 ± 15.86	32.79 ± 13.39	32.56 ± 14.45
Sodium, mmol/L	139.39 ± 3.37	138.88 ± 3.95	137.89 ± 4.64
Potassium, mmol/L	4.42 ± 0.65	4.46 ± 0.65	4.41 ± 0.79
PT, s	19.15 ± 8.15	18.48 ± 2.30	18.66 ± 2.21
APTT, s	16.24 ± 8.84	16.06 ± 7.06	17.52 ± 9.02

1) *: Comparison with Outpatient Group, $p<0.05$; #: Comparison with Emergency Group, $p<0.05$;
2) Transferred, Transferred from Other Medical Institutions Group; SBP, Systolic Blood Pressure; DBP, Diastolic Blood Pressure; LVEF, Left Ventricular Ejection Fraction; LVFS, Left Ventricular Fractional Shortening; IVRT, Isovolumic Relaxation Time; RVSP, Right Ventricular Systolic Pressure; BNP, B-type Natriuretic Peptide; CKMB, Creatine Kinase MB; cTnI, Cardiac Troponin I; RBC, Red Blood Cells; WBC, White Blood Cells; Hb, Hemoglobin; PLT, Platelets; MCV, Mean Corpuscular Volume; MCH, Mean Corpuscular Hemoglobin; MCHC, Mean Corpuscular Hemoglobin Concentration; ALT, Alanine Aminotransferase; AST, Aspartate Aminotransferase; GTP, Gamma-Glutamyl Transpeptidase; ALB, Albumin; LDL, Low-Density Lipoprotein; HDL, High-Density Lipoprotein; TG, Triglycerides; ChE, Cholinesterase; Cr, Creatinine; PT, Prothrombin Time; APTT, Activated Partial Thromboplastin Time

Supplementary Table 3. Comparison of Different Outcomes

Variables	Hospitalization Outcome		Follow-up outcomes	
	Survival (n = 201)	Death (n = 11)	Survival (n = 169)	Death (n = 43)
**Demographic Characteristics**				
Age, years	0.83 (0.33, 7.00)	1.58 (0.38, 5.50)*	1.33 (0.33, 8.00)	0.50 (0.25, 2.83)*
Sex				
Girl	71 (35.32%)	3 (27.27%)	61 (36.09%)	13 (30.23%)
Boy	130 (64.68%)	8 (72.73%)	108 (63.91%)	30 (69.77%)
Mode of delivery				
Normal delivery	103 (51.24%)	6 (54.55%)	91 (53.85%)	18 (41.86%)
Cesarean section	98 (48.76%)	5 (45.45%)	78 (46.15%)	25 (58.14%)
Caregiver				
Others	13 (6.47%)	1 (9.09%)	13 (7.69%)	1 (2.33%)
Parents	188 (93.53%)	10 (90.91%)	156 (92.31%)	42 (97.67%)
Caregiver's education				
High school and below	158 (78.61%)	9 (81.82%)	133 (78.70%)	34 (79.07%)
University and above	43 (21.39%)	2 (18.18%)	36 (21.30%)	9 (20.93%)
Admission route				
Outpatient	105 (52.24%)	6 (54.55%)	97 (57.40%)	14 (32.56%) *
Emergency	38 (18.91%)	2 (18.18%)	29 (17.16%)	11 (25.58%) *
Transferred	58 (28.86%)	3 (27.27%)	43 (25.44%)	18 (41.86%) *
**Clinical features**				
Weight, kg	15.13 ± 13.50	12.50 ± 8.62	16.04 ± 14.01	10.89 ± 8.96*
Height, cm	85.70 ± 34.13	87.00 ± 29.19	88.24 ± 35.32	76.03 ± 25.27*
Respiration, bpm	38.90 ± 14.64	39.45 ± 15.01	37.63 ± 14.57	44.05 ± 13.82*
Heart rate, bpm	122.55 ± 29.64	125.09 ± 27.50	119.97 ± 29.63	133.33 ± 26.62*
Blood pressure				
SBP, mmHg	99.01 ± 16.59	92.82 ± 7.45*	99.41 ± 16.13	95.86 ± 16.82
DBP, mmHg	59.63 ± 13.89	57.55 ± 7.39	59.66 ± 13.69	58.98 ± 13.54
Modified ROSS classification				
I~II	83 (41.29%)	3 (27.27%)	78 (46.15%)	8 (18.60%) *
III~IV	118 (58.71%)	8 (72.73%)	91 (53.85%)	35 (81.40%) *

**Diagnostic-related**				
Initial Diagnosis	144 (71.64%)	6 (54.55%)	116 (68.64%)	34 (79.07%)
Type of CM				
HCM	160 (79.60%)	9 (81.82%)	136 (80.47%)	33 (76.74%)
RCM	35 (17.41%)	1 (9.09%)	29 (17.16%)	7 (16.28%)
RP-HCM	6 (2.99%)	1 (9.09%)	4 (2.37%)	3 (6.98%)
Primary Combined Disease				
Infection	150 (74.63%)	10 (90.91%)	127 (75.15%)	33 (76.74%)
Heart Failure	105 (52.24%)	4 (36.36%)	86 (50.89%)	23 (53.49%)
Arrhythmia	40 (19.90%)	3 (27.27%)	35 (20.71%)	8 (18.60%)
**Auxiliary examination**				
Electrocardiogram				
ST-T changes	78 (38.81%)	5 (45.45%)	65 (38.46%)	18 (41.86%)
QT prolongation	44 (21.89%)	4 (36.36%)	38 (22.49%)	10 (23.26%)
High PR	17 (8.46%)	1 (9.09%)	12 (7.10%)	6 (13.95%)
Ventricular hypertrophy	65 (32.34%)	1 (9.09%)	53 (31.36%)	13 (30.23%)
Atrial Hypertrophy	41 (20.40%)	1 (9.09%)	36 (21.30%)	6 (13.95%)
Echocardiography				
LVEF, %	62.74 ± 11.42	55.09 ± 18.75*	63.58 ± 11.41	57.47 ± 12.93*
LVFS, %	34.19 ± 10.18	28.54 ± 10.96	34.98 ± 10.56	29.65 ± 7.81*
E/A ratio	1.37 ± 0.49	1.41 ± 0.46	1.36 ± 0.48	1.44 ± 0.53
IVRT,s	69.46 ± 28.58	68.73 ± 35.11	69.27 ± 27.62	70.00 ± 33.61
RVSP, mmHg	28.35 ± 13.61	38.73 ± 20.30	27.99 ± 13.48	32.43 ± 16.27
Valvular Regurgitation	114 (56.72%)	9 (81.82%)	96 (56.80%)	27 (62.79%)
Pericardial effusion	38 (18.91%)	1 (9.09%)	27 (15.98%)	12 (27.91%)
Pulmonary hypertension	37 (18.41%)	5 (45.45%)	28 (16.57%)	14 (32.56%) *
Cardiac Biomarkers				
BNP, pg/ml	1360.69 ± 3926.77	1363.20 ± 1622.39	1268.13 ± 4061.47	1725.09 ± 2810.82
CKMB, pg/ml	74.26 ± 447.20	7.90 ± 7.48	32.26 ± 257.63	222.35 ± 811.44
cTnI,ug/L,	1.75 ± 9.94	0.68 ± 1.33	1.33 ± 6.28	3.12 ± 17.64
Complete Blood Count				
RBC, 10^12/L	4.35 ± 0.67	4.23 ± 0.95	4.37 ± 0.67	4.24 ± 0.74
WBC, 10^9/L	10.65 ± 5.28	10.97 ± 7.02	10.62 ± 5.30	10.87 ± 5.68

Hb, g/L	115.41 ± 18.08	115.00 ± 25.29	116.56 ± 17.93	110.81 ± 19.93
PLT, 10^9/L	343.55 ± 155.03	271.27 ± 117.27	343.94 ± 158.32	323.51 ± 135.71
MCV, fl	82.76 ± 6.70	84.92 ± 5.38	82.96 ± 6.89	82.55 ± 5.66
MCH, pg	26.61 ± 2.55	27.23 ± 1.80	26.76 ± 2.59	26.19 ± 2.19
MCHC, g/L	322.42 ± 15.66	321.45 ± 12.99	323.47 ± 15.03	318.05 ± 16.72*
Biochemical Tests				
ALT, U/L	90.71 ± 355.74	80.06 ± 86.53	91.33 ± 387.16	85.52 ± 69.71
AST, U/L	111.82 ± 221.75	127.65 ± 123.17	101.75 ± 227.56	155.41 ± 168.38
GTP, U/L	57.30 ± 74.24	32.65 ± 21.83	57.39 ± 78.44	50.64 ± 43.13
ALB, g/L	40.56 ± 6.54	37.97 ± 7.16	41.13 ± 6.63	37.66 ± 5.65*
LDL, mmol/L	2.24 ± 0.92	3.00 ± 1.90	2.22 ± 0.93	2.52 ± 1.25
HDL, mmol/L	0.99 ± 0.35	1.14 ± 0.80	1.02 ± 0.37	0.93 ± 0.46
TG, mmol/L	1.36 ± 0.74	1.27 ± 0.72	1.40 ± 0.75	1.18 ± 0.69
ChE, U/L	6290.73 ± 1856.90	6214.82 ± 2226.56	6378.32 ± 1850.56	5927.07 ± 1933.10
Cr, umol/L	33.10 ± 14.76	41.62 ± 17.36	32.81 ± 15.12	36.40 ± 14.23
Sodium, mmol/L	138.95 ± 3.91	137.15 ± 3.76	139.05 ± 4.01	138.12 ± 3.45
Potassium, mmol/L	4.45 ± 0.66	4.01 ± 1.10	4.46 ± 0.67	4.27 ± 0.75
PT, s	18.85 ± 6.24	19.39 ± 1.73	18.79 ± 6.73	19.23 ± 2.21
APTT, s	16.43 ± 8.46	19.19 ± 10.54	16.11 ± 8.44	18.37 ± 8.94

1) *: $P < 0.05$

2) HCM, Hypertrophic Cardiomyopathy; RCM, Restrictive Cardiomyopathy; RP-HCM, Hypertrophic cardiomyopathy with restrictive phenotype; Transferred, Transferred from Other Medical Institutions Group; SBP, Systolic Blood Pressure; DBP, Diastolic Blood Pressure; LVEF, Left Ventricular Ejection Fraction; LVFS, Left Ventricular Fractional Shortening; IVRT, Isovolumic Relaxation Time; RVSP, Right Ventricular Systolic Pressure; BNP, B-type Natriuretic Peptide; CKMB, Creatine Kinase MB; cTnI, Cardiac Troponin I; RBC, Red Blood Cells; WBC, White Blood Cells; Hb, Hemoglobin; PLT, Platelets; MCV, Mean Corpuscular Volume; MCH, Mean Corpuscular Hemoglobin; MCHC, Mean Corpuscular Hemoglobin Concentration; ALT, Alanine Aminotransferase; AST, Aspartate Aminotransferase; GTP, Gamma-Glutamyl Transpeptidase; ALB, Albumin; LDL, Low-Density Lipoprotein; HDL, High-Density Lipoprotein; TG, Triglycerides; ChE, Cholinesterase; Cr, Creatinine; PT, Prothrombin Time; APTT, Activated Partial Thromboplastin Time.

Supplementary Table 4. Logistic Regression Analysis of In-Hospital Mortality Risk

Variables	Univariate		Multiple	
	OR (95%CI)	*P*	OR (95%CI)	*P*
Sex				
Girl	1.00 (Reference)			
Boy	1.46 (0.37 ~ 5.66)	0.587		
Mode of delivery				
Normal delivery	1.00 (Reference)			
Cesarean section	0.88 (0.26 ~ 2.96)	0.831		
Caregiver				
Others	1.00 (Reference)			
Parents	0.69 (0.08 ~ 5.83)	0.734		
Caregiver's education				
High school and below	1.00 (Reference)			
University and above	0.82 (0.17 ~ 3.92)	0.8		
Admission route				
Outpatient	1.00 (Reference)			
Emergency	0.92 (0.18 ~ 4.76)	0.922		
Transferred	0.91 (0.22 ~ 3.75)	0.891		
Initial Diagnosis				
No	1.00 (Reference)			
Yes	0.48 (0.14 ~ 1.62)	0.234		
Type of CM				
HCM	1.00 (Reference)			
RCM	0.51 (0.06 ~ 4.14)	0.527		
RP-HCM	2.96 (0.32 ~ 27.31)	0.338		
Modified ROSS classification				
I~II	1.00 (Reference)			
III~IV	1.88 (0.48 ~ 7.28)	0.363		
Infection				
No	1.00 (Reference)			
Yes	3.40 (0.42 ~ 27.21)	0.249		
Arrhythmia				
No	1.00 (Reference)			
Yes	1.51 (0.38 ~ 5.95)	0.556		
Heart Failure				
No	1.00 (Reference)			
Yes	0.52 (0.15 ~ 1.84)	0.312		
Valvular Regurgitation				
No	1.00 (Reference)			
Yes	3.43 (0.72 ~ 16.30)	0.12		

Pericardial effusion				
No	1.00 (Reference)			
Yes	0.43 (0.05 ~ 3.45)	0.426		
Pulmonary hypertension				
No	1.00 (Reference)			
Yes	3.69 (1.07 ~ 12.75)	0.039		
ST-T changes				
No	1.00 (Reference)			
Yes	1.31 (0.39 ~ 4.45)	0.661		
QT prolongation				
No	1.00 (Reference)			
Yes	2.04 (0.57 ~ 7.28)	0.273		
High PR				
No	1.00 (Reference)			
Yes	1.08 (0.13 ~ 8.97)	0.942		
Ventricular hypertrophy				
No	1.00 (Reference)			
Yes	0.21 (0.03 ~ 1.67)	0.14		
Atrial Hypertrophy				
No	1.00 (Reference)			
Yes	0.39 (0.05 ~ 3.14)	0.376		
Age, years	0.99 (0.86 ~ 1.13)	0.84		
Respiration, bpm	1.00 (0.96 ~ 1.04)	0.902		
Heart rate, bpm	1.00 (0.98 ~ 1.02)	0.78		
Weight, kg	0.98 (0.93 ~ 1.04)	0.525		
Height, cm	1.00 (0.98 ~ 1.02)	0.901		
SBP, mmHg	0.97 (0.93 ~ 1.02)	0.214		
DBP, mmHg	0.99 (0.94 ~ 1.04)	0.62		
RBC, 10^12/L	0.77 (0.32 ~ 1.86)	0.566		
WBC, 10^9/L	1.01 (0.91 ~ 1.13)	0.849		
Hb, g/L	1.00 (0.97 ~ 1.03)	0.942		
PLT, 10^9/L	1.00 (0.99 ~ 1.00)	0.125		
MCV, fl	1.05 (0.96 ~ 1.16)	0.292		
MCH, pg	1.11 (0.86 ~ 1.44)	0.428		
MCHC, g/L	1.00 (0.96 ~ 1.04)	0.841		
BNP, pg/ml	1.00 (1.00 ~ 1.00)	0.998		
CKMB, pg/ml	1.00 (0.96 ~ 1.03)	0.799		
cTnI,ug/L,	0.97 (0.84 ~ 1.13)	0.739		
ALT, U/L	1.00 (1.00 ~ 1.00)	0.921		
AST, U/L	1.00 (1.00 ~ 1.00)	0.815		
GTP, U/L	0.99 (0.97 ~ 1.01)	0.258		
ALB, g/L	0.95 (0.87 ~ 1.03)	0.207		
LDL,mmol/L	1.77 (1.10 ~ 2.85)	0.019	2.15 (1.28 ~ 3.61)	0.004
HDL, mmol/L	2.16 (0.61 ~ 7.64)	0.233		
TG,mmol/L	0.83 (0.35 ~ 2.00)	0.681		

ChE, U/L	1.00 (1.00 ~ 1.00)	0.896		
Cr, umol/L	1.03 (1.00 ~ 1.06)	0.081		
Sodium, mmol/L	0.90 (0.79 ~ 1.03)	0.134		
Potassium, mmol/L	0.43 (0.19 ~ 0.98)	0.046		
PT, s	1.01 (0.94 ~ 1.09)	0.78		
APTT, s	1.03 (0.97 ~ 1.09)	0.304		
LVEF, %	0.95 (0.91 ~ 0.99)	0.043	0.95 (0.90 ~ 0.99)	0.026
LVFS, %	0.92 (0.86 ~ 0.99)	0.043		
E/A ratio	1.20 (0.35 ~ 4.09)	0.774		
IVRT, s	1.00 (0.98 ~ 1.02)	0.935		
RVSP, mmHg	1.03 (1.01 ~ 1.06)	0.043	1.04 (1.01 ~ 1.07)	0.01

1) OR: Odds Ratio, CI: Confidence Interval
2) HCM, Hypertrophic Cardiomyopathy; RCM, Restrictive Cardiomyopathy; RP-HCM, Hypertrophic cardiomyopathy with restrictive phenotype; Transferred, Transferred from Other Medical Institutions Group; SBP, Systolic Blood Pressure; DBP, Diastolic Blood Pressure; LVEF, Left Ventricular Ejection Fraction; LVFS, Left Ventricular Fractional Shortening; IVRT, Isovolumic Relaxation Time; RVSP, Right Ventricular Systolic Pressure; BNP, B-type Natriuretic Peptide; CKMB, Creatine Kinase MB; cTnI, Cardiac Troponin I; RBC, Red Blood Cells; WBC, White Blood Cells; Hb, Hemoglobin; PLT, Platelets; MCV, Mean Corpuscular Volume; MCH, Mean Corpuscular Hemoglobin; MCHC, Mean Corpuscular Hemoglobin Concentration; ALT, Alanine Aminotransferase; AST, Aspartate Aminotransferase; GTP, Gamma-Glutamyl Transpeptidase; ALB, Albumin; LDL, Low-Density Lipoprotein; HDL, High-Density Lipoprotein; TG, Triglycerides; ChE, Cholinesterase; Cr, Creatinine; PT, Prothrombin Time; APTT, Activated Partial Thromboplastin Time.

Supplementary Table 5. Cox Regression Analysis of Mortality Risk During Follow-Up

Variables	Univariate		Multiple	
	OR (95%CI)	*P*	OR (95%CI)	*P*
Sex				
Girl	1.00 (Reference)			
Boy	1.18 (0.62 ~ 2.27)	0.61		
Mode of delivery				
Normal delivery	1.00 (Reference)			
Cesarean section	1.66 (0.90 ~ 3.06)	0.104		
Caregiver				
Others	1.00 (Reference)			
Parents	3.23 (0.44 ~ 23.46)	0.247		
Caregiver's education				
High school and below	1.00 (Reference)			
University and above	1.13 (0.54 ~ 2.35)	0.751		
Admission route				
Outpatient	1.00 (Reference)			
Emergency	2.58 (1.17 ~ 5.69)	0.019		
Transferred	2.48 (1.23 ~ 4.99)	0.011		
Initial Diagnosis				
No	1.00 (Reference)			
Yes	1.69 (0.81 ~ 3.53)	0.161		
Type of CM				
HCM	1.00 (Reference)			
RCM	0.86 (0.38 ~ 1.95)	0.719		
RP-HCM	1.89 (0.58 ~ 6.16)	0.294		
Modified ROSS classification				
I~II	1.00 (Reference)			
III~IV	3.75 (1.73 ~ 8.15)	<.001		
Infection				
No	1.00 (Reference)			
Yes	1.30 (0.64 ~ 2.66)	0.466		
Arrhythmia				
No	1.00 (Reference)			
Yes	0.85 (0.39 ~ 1.83)	0.674		
Heart Failure				
No	1.00 (Reference)			
Yes	1.06 (0.58 ~ 1.93)	0.847		
Valvular Regurgitation				
No	1.00 (Reference)			
Yes	1.07 (0.57 ~ 1.99)	0.841		
Pericardial effusion				
No	1.00 (Reference)			
Yes	1.83 (0.94 ~ 3.56)	0.077		
Pulmonary hypertension				

No	1.00 (Reference)		1.00 (Reference)	
Yes	2.05 (1.08 ~ 3.88)	0.028	2.40 (1.24 ~ 4.64)	0.009
ST-T changes				
No	1.00 (Reference)			
Yes	1.09 (0.59 ~ 2.00)	0.78		
QT prolongation				
No	1.00 (Reference)			
Yes	0.91 (0.45 ~ 1.85)	0.795		
High PR				
No	1.00 (Reference)			
Yes	2.42 (1.01 ~ 5.75)	0.046		
Ventricular hypertrophy				
No	1.00 (Reference)			
Yes	0.92 (0.48 ~ 1.77)	0.807		
Atrial Hypertrophy				
No	1.00 (Reference)			
Yes	0.57 (0.24 ~ 1.36)	0.208		
Age, years	0.85 (0.78 ~ 0.94)	0.001		
Respiration, bpm	1.03 (1.01 ~ 1.06)	<.001		
Heart rate, bpm	1.02 (1.01 ~ 1.03)	<.001		
Weight, kg	0.94 (0.90 ~ 0.97)	0.001	0.92 (0.88 ~ 0.96)	<.001
Height, cm	0.98 (0.97 ~ 0.99)	0.003		
SBP, mmHg	0.98 (0.96 ~ 1.00)	0.067		
DBP, mmHg	0.99 (0.97 ~ 1.02)	0.528		
RBC, 10^12/L	0.70 (0.45 ~ 1.08)	0.104		
WBC, 10^9/L	1.01 (0.95 ~ 1.06)	0.825		
Hb, g/L	0.98 (0.96 ~ 0.99)	0.023		
PLT, 10^9/L	1.00 (1.00 ~ 1.00)	0.801		
MCV, fl	0.99 (0.95 ~ 1.04)	0.779		
MCH, pg	0.94 (0.84 ~ 1.04)	0.234		
MCHC, g/L	0.98 (0.96 ~ 0.99)	0.044		
BNP, pg/ml	1.00 (1.00 ~ 1.00)	0.544		
CKMB, pg/ml	1.01 (1.01 ~ 1.01)	0.013	1.01 (1.01 ~ 1.01)	0.005
cTnI,ug/L,	1.01 (0.99 ~ 1.03)	0.267		
ALT, U/L	1.00 (1.00 ~ 1.00)	0.918		
AST, U/L	1.27 (0.96 ~ 1.66)	0.091		
GTP, U/L	0.47 (0.19 ~ 1.16)	0.101		
ALB, g/L	0.67 (0.42 ~ 1.07)	0.095	0.94 (0.90 ~ 0.98)	0.007
LDL,mmol/L	1.00 (1.00 ~ 1.00)	0.157	1.48 (1.11 ~ 1.96)	0.007
HDL, mmol/L	1.01 (0.99 ~ 1.02)	0.49		
TG,mmol/L	0.95 (0.89 ~ 1.02)	0.143	0.52 (0.31 ~ 0.87)	0.013
ChE, U/L	0.72 (0.46 ~ 1.11)	0.136		
Cr, umol/L	1.01 (0.97 ~ 1.04)	0.681		
Sodium, mmol/L	0.94 (0.90 ~ 0.98)	0.003		
Potassium, mmol/L	1.00 (0.99 ~ 1.00)	0.62		
PT, s	0.97 (0.95 ~ 0.99)	0.004		

APTT, s	1.33 (0.74 ~ 2.38)	0.344		
LVEF, %	1.00 (0.99 ~ 1.01)	0.806	0.96 (0.93 ~ 0.98)	<.001
LVFS, %	1.01 (0.98 ~ 1.05)	0.365		
E/A ratio	1.00 (1.00 ~ 1.00)	0.166		
IVRT, s	1.01 (1.00 ~ 1.03)	0.083		
RVSP, mmHg	0.94 (0.91 ~ 0.98)	<.001		

1) OR: Odds Ratio, CI: Confidence Interval
2) HCM, Hypertrophic Cardiomyopathy; RCM, Restrictive Cardiomyopathy; RP-HCM, Hypertrophic cardiomyopathy with restrictive phenotype; Transferred, Transferred from Other Medical Institutions Group; SBP, Systolic Blood Pressure; DBP, Diastolic Blood Pressure; LVEF, Left Ventricular Ejection Fraction; LVFS, Left Ventricular Fractional Shortening; IVRT, Isovolumic Relaxation Time; RVSP, Right Ventricular Systolic Pressure; BNP, B-type Natriuretic Peptide; CKMB, Creatine Kinase MB; cTnI, Cardiac Troponin I; RBC, Red Blood Cells; WBC, White Blood Cells; Hb, Hemoglobin; PLT, Platelets; MCV, Mean Corpuscular Volume; MCH, Mean Corpuscular Hemoglobin; MCHC, Mean Corpuscular Hemoglobin Concentration; ALT, Alanine Aminotransferase; AST, Aspartate Aminotransferase; GTP, Gamma-Glutamyl Transpeptidase; ALB, Albumin; LDL, Low-Density Lipoprotein; HDL, High-Density Lipoprotein; TG, Triglycerides; ChE, Cholinesterase; Cr, Creatinine; PT, Prothrombin Time; APTT, Activated Partial Thromboplastin Time.
